# Subalpine Pyrenees received higher nitrogen deposition than predicted by EMEP and CHIMERE chemistry-transport models

**DOI:** 10.1038/srep12942

**Published:** 2015-08-10

**Authors:** Marion Boutin, Thierry Lamaze, Florian Couvidat, André Pornon

**Affiliations:** 1 Université Toulouse 3 Paul Sabatier, CNRS, ENFA, UMR5174 Laboratoire Evolution & Diversité Biologique, 118 route de Narbonne 31062 Toulouse Cedex 9, France.; 2 Centre d’Etudes Spatiales de la BIOsphère, 31401 Toulouse Cedex 9, France.; 3 INERIS, Institut National de l’Environnement Industriel et des Risques, 60550 Verneuil-en-Halatte, France.

## Abstract

Deposition of reactive nitrogen (N) from the atmosphere is expected to be the third greatest driver of biodiversity loss by the year 2100. Chemistry-transport models are essential tools to estimate spatially explicit N deposition but the reliability of their predictions remained to be validated in mountains. We measured N deposition and air concentration over the subalpine Pyrenees. N deposition was found to range from 797 to 1,463 mg N m^−2^ year^−1^. These values were higher than expected from model predictions, especially for nitrate, which exceeded the estimations of EMEP by a factor of 2.6 and CHIMERE by 3.6. Our observations also displayed a reversed reduced-to-oxidized ratio in N deposition compared with model predictions. The results highlight that the subalpine Pyrenees are exposed to higher levels of N deposition than expected according to standard predictions and that these levels exceed currently recognized critical loads for most high-elevation habitats. Our study reveals a need to improve the evaluation of N deposition in mountains which are home to a substantial and original part of the world’s biodiversity.

Half the reactive nitrogen (N) produced annually on earth results from anthropogenic activities^1^. For decades, anthropogenic N emissions have deeply altered the composition of the earth’s atmosphere and atmospheric deposition, with diverse human and environmental repercussions^2^. It is thought that by the year 2100 atmospheric nitrogen deposition will be the third greatest driver of biodiversity loss^3^. Recent studies have stressed that long-term chronic enhancement of N deposition may have a detrimental effect on plant communities even at low levels^4^,^5^,^6^,^7^. Therefore, many biodiversity hot-spots and priority conservation areas on the planet are potentially threatened by chronic, long-range N pollution^8^. To assess this threat and orientate conservation policies, reliable spatially explicit estimations of the amounts and forms of atmospheric N deposition are crucially needed.

Impelled by the creation of the Convention on Long-Range Transboundary Air Pollution (CLRTAP) in 1979, modelling tools were developed to simulate pollutant transport and deposition over Europe. Among them, the eulerian chemistry-transport models EMEP (European Monitoring and Evaluation Programme)^9^ and CHIMERE^10^ were developed to simulate air quality at a regional scale. Those models can also be used to simulate spatio-temporal N deposition and are currently used to assess the impacts of N deposition on plant communities along large spatial^7^ and temporal gradients^11^, and also to estimate exceedances of N critical loads^12^. However, despite the constant improvement of model parameterizations and the quality of input data, simulations still suffer from the lack of and the uneven distribution of measurement stations^13^. Although modelled data have been validated in several well monitored regions^14^, a lack of reliability of deposition estimates is still to be suspected in regions distant from any measurement stations, especially with complex orography and meteorology. Mountain regions typically cumulate both these characteristics. Moreover, remote high-elevation sites are rarely included in large measurement networks and studies involving local and punctual measurements at mountain sites are scarce^15^. Preservation of high elevation habitats is a priority both in terms of biodiversity conservation and water quality insurance. Therefore it is of primary importance to collect complementary N deposition data in mountains and to compare them with chemistry-transport simulations for these regions.

We equipped eight sites above the treeline (between 1,500 and 2,000 m a.s.l.) in the Pyrenees along a 100 km geographical transect (see Supplementary Fig. S1). At each site, we measured bulk N (NO_3_^−^ and NH_4_^+^) deposition and air gaseous NO_2_ and NH_3_ concentrations during the growing season from June to October and bulk N deposition in snow during winter from November to May. Then we compared our observations with the values from the 2012–2013 years for the two models.

## Results

**Figure 1:**
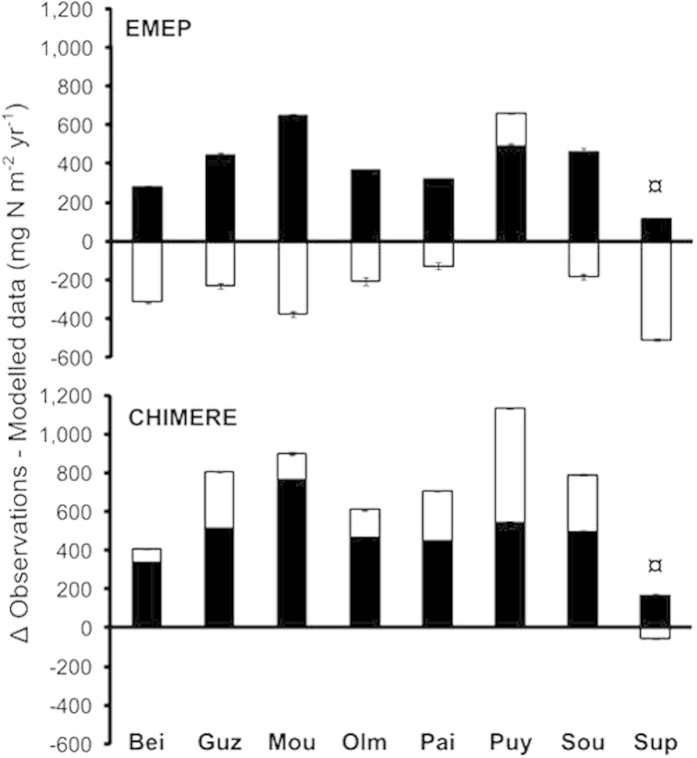
Discrepancy between N deposition observations and modelled data. Difference between bulk measurements (rain + snow) and EMEP (upper panel) or CHIMERE (lower panel) total (wet + dry) modelled values for reduced N (white) and oxidized N (black). Mean of the difference between observations and each year of the modelled values, the error bars represent the value of each year individually. ¤ The *Sup* site observations only take into account bulk rain measurements (June-October).

**Figure 2:**
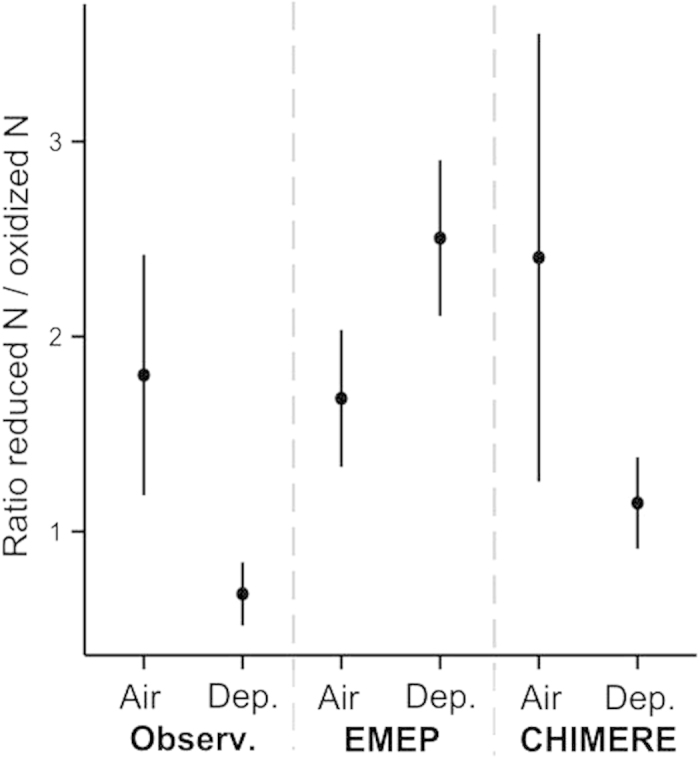
Reduced-to-oxidized ratios in air and deposition. Mean ratio (reduced/oxidized N forms) in air and in total deposition (Dep.). The error bars represent 95% confidence intervals.

Measured NO_3_^−^ deposition (705 ± 128 mg N m^−2^ year^−1^; mean ± sd; Table 1) was on average 2.6-fold higher than EMEP values (paired Wilcoxon-rank-sum-test, n = 7, P = 0.016; Fig. 1) and 3.6-fold higher than CHIMERE values (n = 7, P = 0.016; Fig. 1). Measured NH_4_^+^ deposition (478 ± 139 mg N m^−2^ year^−1^; Table 1) was significantly 1.5-fold lower than from EMEP data (n = 7, P = 0.047; Fig. 1) but was on average 2.4-fold higher than from CHIMERE values (n = 7, P = 0.016; Fig. 1). These results are supported by the relatively high error metrics, especially for NO_3_^−^ (Mean Normalized Bias of −60% for EMEP and −71% for CHIMERE; Table 2). Overall, observed NO_3_^−^ + NH_4_^+^ deposition was higher than EMEP simulations by 295 mg N m^−2^ year^−1^ on average (n = 7, P = 0.031) and higher than CHIMERE simulations by 763 mg N m^−2^ year^−1^ on average (n = 7, P = 0.016; Fig. 1). Finally, as a notable consequence of the strong discrepancy between observed and modelled values for nitrate (oxidized N) deposition, ratios of reduced to oxidized N exceeded 1 (2.5 ± 0.5 for EMEP and 1.15 ± 0.3 for CHIMERE) when calculated from modelled data while they were below 1 (0.7 ± 0.4) when calculated from observations (Fig. 2).

**Figure 3:**
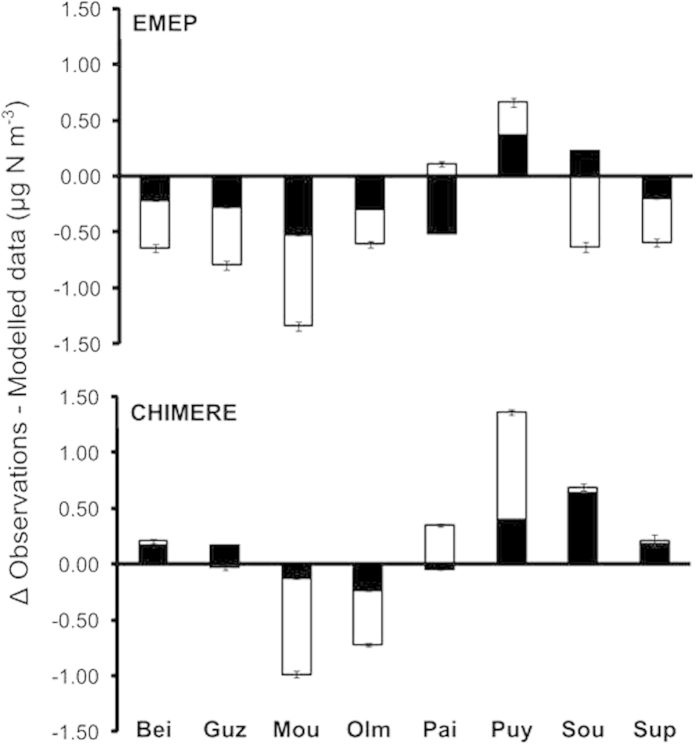
Discrepancy between N air concentration observations and modelled data. Difference between passive gas sampler measurements and EMEP (upper panel) or CHIMERE (lower panel) modelled values for NH_3_ (white) and NO_2_ (black) air concentrations. Mean of the difference between observation and each year of the modelled values, the error bars represent the value of each year individually.

Average measured NO_2_ concentration in air (0.48 μg N m^−3^ ± 0.27; Table 1) was not significantly different from EMEP (n = 8, P = 0.207; Fig. 3) nor from CHIMERE predictions (n = 8, P = 0.233; Fig. 3), and measured NH_3_ air concentration (0.75 μg N m^−3^ ± 0.26; Table 1) was significantly lower than from EMEP (n = 8, P = 0.039; Fig. 3) and not significantly different from CHIMERE data (n = 8, P = 0.641; Fig. 3). Error metrics were still relatively high (Table 2), because the magnitude of the difference between modelled and observed concentrations was high relative to the low concentrations observed or expected. In contrast to deposition, observed ratios of reduced to oxidized N air concentrations (1.8 ± 0.3) and modelled ratios (1.68 ± 0.42 for EMEP and 2.72 ± 1.32 for CHIMERE) were greater than 1, indicating higher concentrations of NH_3_ than of NO_2_ in air (Fig. 2).

## Discussion

N deposition was evaluated with a conventional resin-based bulk deposition method. The winter devices might have collected both wet and dry deposition over the snow pack during the period they were fully covered with snow. The summer devices collected wet deposition but only part of the dry deposition since the surface of the collectors was not entirely representative of the rough exchange surface of vegetation which allows plants to catch N from aerosols, gaseous and occult (fog) deposition^16^. Therefore, the deposition measured in this study could be closer to total deposition (wet + dry) than to wet deposition, but cannot be actually considered as total deposition. Since we compared these observed deposition values with the total (wet + dry) deposition values from the models, the approximations made here should have led to the observed values being lower than the modelled ones, not consistently higher as noted in the present work. Thus, the underestimation made by the simulations could thus even be greater than observed here. Especially, fog water has been shown to be more concentrated in N ions than precipitation and to make a large contribution to total N deposition at high elevation^17^,^18^ but is difficult to simulate in modelling^19^. The difference between N compounds included in modelled total deposition and in measured bulk deposition may on the other hand explain part of the difference observed in the ratios of reduced to oxidized N. Especially, oxidized N deposition can be composed of a large variety of compounds (NO_x_, HNO_3_ in gas and particulates, HONO, PAN organic molecules, organonitrate…).

Few measurements of N deposition in European high-elevation open areas are available. Recent studies in different regions of the Alps found levels of deposition in a range similar to those observed in this study (between 500 and 1,360 mg N m^−2^ year^−1^)^20^,^21^. Measurements of bulk deposition by sampling water from rain and snow events on four sites in the Spanish Pyrenees in 1987–1988 led to estimations of N deposition between 391 and 1,041 mg N m^−2^ year^−1^ 22. In the Swiss Alps, NH_3_ concentrations ranging from 0.5 to 1.1 μg N m^−3^ have been observed between April and September at elevations ranging from 1,070 to 1,914 m^23^; and in the Austrian Alps, NH_3_ concentrations ranging from 0.2 to 2.5 μg N m^−3^ (0.7 μg N m^−3^ on average) have been observed over a year between 930 and 1,758 m of elevation^24^. These results stress that the values of N deposition or concentration observed in the present study are not unusual in high-elevation areas of Europe. However, the differences observed between the studies could result from both the methods used for the measurements, specific climatic conditions and different chemical origins and pathways for each region.

By comparing our measurements with estimations made by EMEP and CHIMERE, we found consistently higher values of N deposition (and more particularly of oxidized N) than predicted by these chemistry-transport models.

Part of the discrepancy between observation and modelled data could be suspected to come from incommensurability, *i.e.* the difference between spatial representations of measurements and simulations^25^: simulations tend to provide averaged values representative of a relatively vast area (2500 km^2^ in the case of EMEP, 16 km^2^ for CHIMERE), whereas measurements are conducted at a very local scale and cannot be reasonably considered as representative of large areas, especially in such heterogeneous topography as mountain landscapes.

In the study region, precipitation amounts during the measurements period (from June 2012 to May 2013) were similar to average precipitation received over the two calendar years 2012 and 2013. The difference was of only 66 mm more in June 2012 – May 2013 than the average precipitation amount over 2012–2013. However, it was of 402 mm more than in 2012 and 269 mm less than in 2013 (see Supplementary Table S2).

Finally, we did not find an underestimation of gaseous N concentration in the air. On the contrary, observed values even tended to be lower than expected by EMEP, especially in the case of NH_3_. Lower values might come from slightly less efficient sampling by the passive samplers in field conditions (low concentrations of the targeted gases) compared with the lab conditions they were tested in. Moreover, we did not measure N aerosol and other gaseous oxidized N species concentrations (among which HNO_3_ is expected to be an important contributor to deposition). Thus, an underprediction of the concentration of these aerosols or other gases in the atmosphere by the models might still account for the discrepancy between modelled *vs.* measured deposition.

Studies comparing EMEP and CHIMERE predictions with measured N deposition found that these models performed rather well in low elevation regions. Indeed, in north-western Europe, EMEP globally underpredicted N deposition by only 10% when compared with the ICP-forests and EMEP/CCC measurement networks^14^. In Spain, models slightly underestimated N deposition when compared with the ICP-forests and Catalan Air Quality networks^12^, but in this study bulk deposition measurements were compared with model predictions for wet only (and not wet + dry) deposition, which may explain the discrepancy. Therefore, the underestimation observed in our study might be specific to the high elevation nature of this area.

Because a thorough evaluation of precipitation is crucial to accurately model deposition amounts^14^, the underestimation of deposition observed here could result from an underestimation of precipitation amounts by the models. However, on average over 2012 to 2013, EMEP and CHIMERE underestimated precipitation amounts only by a factor of 0.95 and 0.76 respectively in our study area (see Supplementary Table S2). The cause of the discrepancy in N deposition could thus be rather the particularity of deposition events at high elevation, especially snow and occult deposition. Indeed, as EMEP and CHIMERE models are not specific to mountain regions, they might lack input data and parameters to correctly simulate snow and occult deposition which represent a large part of total N deposition at high elevation^17^,^18^,^26^. In that case, the discrepancy between model predictions and measurements observed in the Pyrenees might occur in other mountain regions.

Finally, it cannot be excluded that the discrepancy might come from an underestimation in the N emissions data used in these models.

Our findings suggest that the subalpine zone of the Pyrenees could be more seriously threatened by N deposition than predicted by EMEP and CHIMERE simulations. Indeed, while the currently recognized critical loads for N in subalpine grasslands are 500–1,000 mg N m^−2^ year^−1^ 27, all 8 sites studied here received a N load of over 500 mg N m^−2^ year^−1^ and 6 of them received more than 1,000 mg N m^−2^ year^−1^ (Table 1). According to EMEP data, 8 sites were predicted to receive a N load of over 500 mg N m^−2^ year^−1^ but only one or two sites (in 2012 or 2013 respectively) were predicted to receive a N load of over 1,000 mg N m^−2^ year^−1^ (see Supplementary Table S1). According to CHIMERE, none or 1 site (in 2012 or 2013 respectively) was predicted to receive a N load of over 500 mg N m^−2^ year^−1^, and no sites were predicted to receive a N load of over 1,000 mg N m^−2^ year^−1^ (see Supplementary Table S1). N availability in high-elevation soils is considered low because of slow N mineralization rates, substantial N competition between microbes and plants, and considerable plant N re-allocation^26^. Such abundant N deposition in these habitats could thus considerably increase N availability and lead to eutrophication. Deposition of reduced N on soils with low buffer capacity can lead to acidification via the processes of nitrification and root uptake which both release H^+^ ions; then soil acidification and leaching of nitrates (from deposition of oxidized N or from nitrification) can cause base cation depletion^28^,^29^. By underestimating NH_4_^+^ deposition, the CHIMERE model could thus also underestimate the risk of soil acidification. Eutrophication and acidification are the main pathways to N deposition mediated biodiversity loss^27^. Many characteristic high-elevation species are typically adapted to nutrient-poor conditions and have a limited ability to respond to an increase in N availability^30^,^31^. Therefore, subalpine biodiversity could be especially at risk as many characteristic species could be outcompeted as a consequence of eutrophication^32^ and because these soils usually have a low buffer capacity^26^.

Our findings also report reversed reduced to oxidized ratios in N deposition in high-elevation Pyrenees compared with those predicted from EMEP or CHIMERE. A misestimation of the ratio between N forms in deposition might lead to wrong predictions of the effects on plant communities as their composition depends on the partitioning of differently available forms of N^33^. In subalpine grasslands, plants preferentially use NH_4_^+^ (the dominant form of N in these soils), but some species, especially certain grasses, are more able to use NO_3_^−^ than others (for instance certain shrubs)^34^. Overall, such species could thus be more able to take advantage of an additional input of NO_3_^−^ and outcompete less competitive species. Furthermore, as about half of the NO_3_^−^ deposition occurs from snow (Table 1), when vegetation uptake is low, and because NO_3_^−^ is highly soluble, high levels of NO_3_^−^ deposition could be a threat to stream and lake water quality^35^,^36^. Such potential environmental impacts could thus be missed if only these simulations are taken as reference.

This study does not aim to challenge the high quality and usefulness of chemistry-transport models such as EMEP and CHIMERE but provides complementary observations from areas crucially lacking such data and provides an alert as to the risk of underestimating nitrogen deposition in this mountain area when working on the basis of these predictions. Our results stress the need to improve the evaluation of N deposition (both through improvement of observations and models) in high-elevation ecosystems which are home to a substantial and original part of world biodiversity.

## Methods

The study was conducted at eight sites in the central part of the French Pyrenees (see Supplementary Fig. S1), in open subalpine habitat areas (between 1,500 and 2,000 m a.s.l.). This region is under a cold sub-oceanic climate with annual precipitation of 1245 mm and annual mean temperature of 9.6 °C on average over the past five years (data from Meteo-France for the stations presented in Supplementary Table S2). The Pyrenean subalpine belt is characterized by a mosaic of extensively grazed grasslands, heathlands and scattered conifers groves.

## Bulk N deposition measurements. 

Bulk N deposition was measured over one year (from June 2012 to May 2013).

From June to October, each site was equipped with two 20 cm diameter HDPE funnels, each connected to a PVC column (1/2” diameter and 30 cm length) filled with 30 g of mixed-bed ion-exchange resin (IONAC® NM-60, Lanxess) and fixed 2 m above ground. A polyester fibre plug was inserted at the connection between the funnel and the column to prevent the entry of insects or large particles into the column. Resin columns were protected from excessive heating by being inserted into 10 cm diameter PVC tubes. This device was adapted from Fenn & Poth (2004)^37^. Because sampled water passes through the resin column without any stagnation, the device limits N loss due to algal or bacterial development. One of the two columns was exposed over periods of one month, the other was exposed during the entire sampling period (five months). Columns were pre-rinsed with 100 mL of deionized water and extracted twice by percolating 200 mL of 2 M KCl. Columns exposed over five months did not show signs of resin saturation, however they were more susceptible to human or animal degradation (one stolen, two degraded by bird droppings). Thus we chose to exploit only the results from the one-month exposure columns. The comparison between the sum of the five one-month exposure columns and the five-month exposure columns allowed us to check for intra-site variability. On average, this variability was of 12% for NO_3_^−^ and 19% for NH_4_^+^.

We employed a method similar to that of Susfalk and Johnson (2002)^38^ and Brooks *et al*. (1996)^39^ to measure winter bulk N deposition. In November, at each site, two HDPE tubes (16 cm diameter and 12 cm length) were installed in the soil at least 1 m apart. The tubes contained 150 g of mixed-bed ion-exchange resin (IONAC® NM-60, Lanxess) in a water-porous bag of the same diameter inserted between two porous polyethylene foam discs to prevent direct contact with snow on the top and soil at the bottom but to allow snow melt water circulation through the device. Tubes protruded 2.5 cm above soil surface to limit potential external contamination and avoid disturbance of the natural deposition and accumulation of snow. They were installed on flat or gently sloping areas to limit potential resin contamination by soil erosion during melting. No trace of soil matter was found inside the devices at their removal. The low vegetation surrounding the tubes and the absence of livestock during the exposure period would have limited the risks of resin contamination by splash during rain events or droppings from animals. Although wild fauna droppings cannot be excluded, it remains improbable that they could have occurred similarly across all the sites. Contaminations might also have occurred from blown soil or organic matter dust but would represent very low amounts as most of the exposure period corresponded to the presence of a snow pack or humid atmospheric and edaphic conditions. Due to low winter temperatures and the absence of water stagnation in our device, microbial contamination was expected to be absent or insignificant. At the end of the sampling period (May), the resins were collected, pre-rinsed with 500 mL of deionized water and extracted twice by stirring for 30 minutes with 500 mL of a 2 M KCl solution. The results of the two bags at each site were averaged. Winter measurements at the *Sup* site were discarded as the sampling devices were not retrieved at the same date as for the other sites because of blocked access to the site after a flooding event. Intra-site variability was on average for the seven sites of 20% for NH_4_^+^ (ranging from 0.3 to 23.5%, with the *Mou* site being an exception at 60%) and 48% for NO_3_^−^ (ranging from 7.9 to 49.7%, with the *Bei*, *Mou* and *Puy* sites presenting exceptionally high variability: respectively 78.4, 70.8 and 80%). This variability may be explained by differences in snow heights and snow melt water pathways as already suggested in other studies^39^,^40^. The greater variability observed for NO_3_^−^ compared to NH_4_^+^ could be consistent with this explanation as phenomena of preferential elution of some ionic species over others at different stages of the melting process have been suggested^41^.

As controls, unexposed capped blank columns were installed on 3 sites during the summer period and hermetically wrapped blank resin bags were installed in the soil at 2 sites during the winter period. Blank resins were installed on and retrieved from the sites at the same time as exposed resins, and were extracted and analyzed in the same way as sampling resins. No detectable concentrations of NO_3_^−^ were found in blank extracts from the columns and NO_3_^−^ in the blank extracts from the winter devices represented 14% ± 13% (mean ± sd) of the total NO_3_^−^ found in exposed resins. Blank values for NH_4_^+^ represented 40% ± 18% of NH_4_^+^ extracted from exposed resins columns and 35% ± 13% of NH_4_^+^ extracted from exposed winter devices. These blank values were retrieved from the sampling results. NH_4_^+^ contamination in the blank resins can arise from the release of quaternary amine compounds from the resin polymer^36^ and from the presence of background levels of N in the KCl salt used in the extraction solution. Extracts were analyzed by colorimetry (Alpkem continuous flow analyzer). For the columns, the first extraction recovered 100% of the total NH_4_^+^ and NO_3_^−^ fixed on the resin (the second extraction did not recover any further N ions). For the bags, the N ions retrieved by the two successive extractions were summed (on average, first extraction recovered 70% of the total N recovered by the two extractions).

## Air gaseous N concentration measurements. 

Air gaseous NO_2_ and NH_3_ were sampled with radial passive diffusive samplers (Radiello®, Supelco Analytical). The limit of detection was 1 μg m^−3^ for 24 hour exposure for NH_3_ and 1 ppb after 7 days exposure for NO_2_. Samplers were exposed over a two-week period each month from June to October 2012 using the device supplied by the manufacturer. Adsorbing cartridges were then extracted in deionized water according to the manufacturer’s recommendations and the extracts analyzed by colorimetry (Alpkem continuous flow analyzer). The results were converted into mean gas concentration in air (in μg N m^−3^) over the sampling period according to the manufacturer’s equations (accounting for NO_2_^−^ or NH_4_^+^ mass found in the cartridge, exposure time, sampling rate and temperature during exposure for NO_2_, measured with data loggers (Lascar EL-USB-2+, Lascar Electronics)). Correction of the sampling rates for atmospheric pressure was considered as negligible^42^. As NO_2_ concentration was first obtained in ppb, the conversion to μg m^−3^ was made taking into account the temperature and atmospheric pressure at the sites^43^. In order to check repeatability, 3 sites were equipped with 3 replicate samplers over each sampling period. On average, variability ranged from 5 to 25% depending on the site. For controls, laboratory blank cartridges from the same batches as those exposed were analyzed.

## Chemistry-transport models. 

The eulerian chemistry-transport models CHIMERE (version 2012) and EMEP (version rv4.5) provided estimates of N deposition. Although they have roughly the same approach, they are based on different technical characteristics (meteorology, emissions and chemical mechanisms).

The CHIMERE model was developed, maintained and distributed by IPSL (CNRS) and INERIS. See Menut *et al*. (2013)^10^ for the model description. More information is available at http://www.lmd.polytechnique.fr/chimere^44^. The CHIMERE model was run over France with a spatial resolution of 4 × 4 km. Boundary conditions for gaseous and particulate species were obtained from nested simulations over Europe. These simulations were conducted with the EMEP emission inventory^45^ for anthropogenic emission and the MEGAN emission model^46^ for biogenic emissions. Meteorology was obtained from the European Centre for Medium-Range Weather Forecasts (ECMWF) model.

The EMEP model was developed at the EMEP Centre MSC-W, hosted by the Norwegian Meteorological Institute. See Simpson *et al*. (2012)^9^ for the model description. Output data and more information are available at http://www.emep.int/47. The EMEP data were retrieved for France at a spatial resolution of 50 × 50 km. Meteorology was simulated from the ECMWF-IFS Cycle 38r2. Emissions were derived from 2012 official data submissions to UNECE CLRTAP^48^.

We compared our observations with the EMEP and CHIMERE data for the 2012 and 2013 calendar years. Inter-annual variability in the modelled data was very low and was negligible compared to the differences between modelled *vs.* observed data, as illustrated by the error bars in Figs 1 and 3. Data available are annual means of NO_2_ and NH_3_ air concentrations (μg N m^−3^) and annual accumulation of dry or wet and reduced or oxidized N (mg N m^−2^; see Supplementary Table S1). In the case of EMEP data, the NH_3_ concentration was not directly available but was calculated as [NH_3_ + NH_4_^+^] minus [Fine NH_4_^+^ particulate matter].

## Data analysis

All analyses were conducted in *R v3.0.2*^49^. Bilinear interpolation of modelled data to match monitored sites was conducted with the packages *rgdal*^50^ and *akima*^51^. Non-parametric paired Wilcoxon-ranked-sum-tests were used to test the significance of the difference between observations and modelled values across the eight sites (averaged between the two years). Deposition values were compared between bulk deposition measurements (rain + snow) and total modelled deposition (wet + dry), excluding the *Sup* site where snow deposition was not accounted for. Error metrics commonly used in model evaluations such as mean normalized absolute error (MNAE) and mean normalized bias (MNB) were calculated.

## Additional Information

**How to cite this article**: Boutin, M. *et al*. Subalpine Pyrenees received higher nitrogen deposition than predicted by EMEP and CHIMERE chemistry-transport models. *Sci. Rep.* **5**, 12942; doi: 10.1038/srep12942 (2015).

## Supplementary Material

Supplementary Information

## Figures and Tables

**Table 1:** Measured N air concentration and bulk deposition.

Site	Airconcentration(μgN m^−3^)		Rain bulkdeposition(mg N m^−2^ year^−1^)		Snow bulkdeposition(mg N m^−2^ year^−1^)		Rain + snow bulkdeposition(mg N m^−2^ year^−1^)	
	NH_3_	NO_2_	NH_4_^+^	NO_3_^−^	NH_4_^+^	NO_3_^−^	NH_4_^+^	NO_3_^−^
*Bei*	0.58	0.37	125	339	142	191	267	530
*Guz*	0.79	0.35	204	373	321	323	526	696
*Mou*	0.50	0.29	161	291	298	665	459	956
*Olm*	0.57	0.38	160	340	249	335	409	675
*Pai*	0.86	0.27	180	296	286	367	466	663
*Puy*	1.32	1.02	174	364	558	367	732	731
*Sou*	0.60	0.78	159	497	330	189	489	685
*Sup*	0.81	0.39	175	355	n.a.	n.a.	n.a.	n.a.

Average NH_3_ and NO_2_ air concentrations (μg N m^−3^), rain, snow and rain + snow cumulated NH_4_^+^ and NO_3_^−^ bulk deposition (mg N m^−2^ year^−1^) measured at the eight sites. n.a. These observations were excluded because the sampling devices were not exposed for the same period of time.

**Table 2:** Error metrics for the comparison of measured and modelled N concentration and deposition.

	MNAE	MNB
**EMEP**		
**NH**_**3**_	69% (±5%)	60% (±7%)
**NO**_**2**_	88% (±1%)	72% (±1%)
**NH**_**4**_^**+**^	55% (±2%)	48% (±2%)
**NO**_**3**_^**−**^	60% (±2%)	−60% (±2%)
**CHIMERE**		
**NH**_**3**_	50% (±1%)	16% (±5%)
**NO**_**2**_	48% (±1%)	−17% (±0%)
**NH**_**4**_^**+**^	49% (±0%)	−49% (±0%)
**NO**_**3**_^**−**^	71% (±1%)	−71% (±1%)

Mean normalized absolute error (MNAE) and Mean normalized bias (MNB) for the comparison of observed and EMEP and CHIMERE values for gaseous N concentrations (NH_3_ and NO_2_); and for the observed bulk deposition (NH_4_^+^ and NO_3_^−^ in rain + snow) with EMEP and CHIMERE modelled values for N deposition (wet + dry). Values are mean (±sd) for the 2 years of modelled data.
