# Supplementary material for: Subalpine Pyrenees received higher nitrogen deposition than predicted by EMEP and CHIMERE chemistry-transport models
Source: Sci Rep. 2015 Aug 10;5:12942. doi: 10.1038/srep12942 (PMC4530447; doi:10.1038/srep12942)
Supplement: Supplementary Information [file srep12942-s1.pdf]

Subalpine Pyrenees received higher nitrogen deposition than predicted by EMEP and CHIMERE chemistry-transport models

Authors: Marion Boutin[1,2*], Thierry Lamaze[2], Florian Couvidat[3] and André Pornon[1]

Affiliations:

[1]Université Toulouse 3 Paul Sabatier, CNRS, ENFA, UMR5174 Laboratoire Evolution & Diversité Biologique, 118 route de Narbonne 31062 Toulouse Cedex 9, France

[2] Centre d'Etudes Spatiales de la BIOsphère, 31401 Toulouse Cedex 9, France

[3] INERIS, Institut National de l’Environnement Industriel et des Risques, 60550 Verneuil-en-Halatte, France

*Correspondence to: marionboutin@free.fr

SUPPLEMENTARY INFORMATION

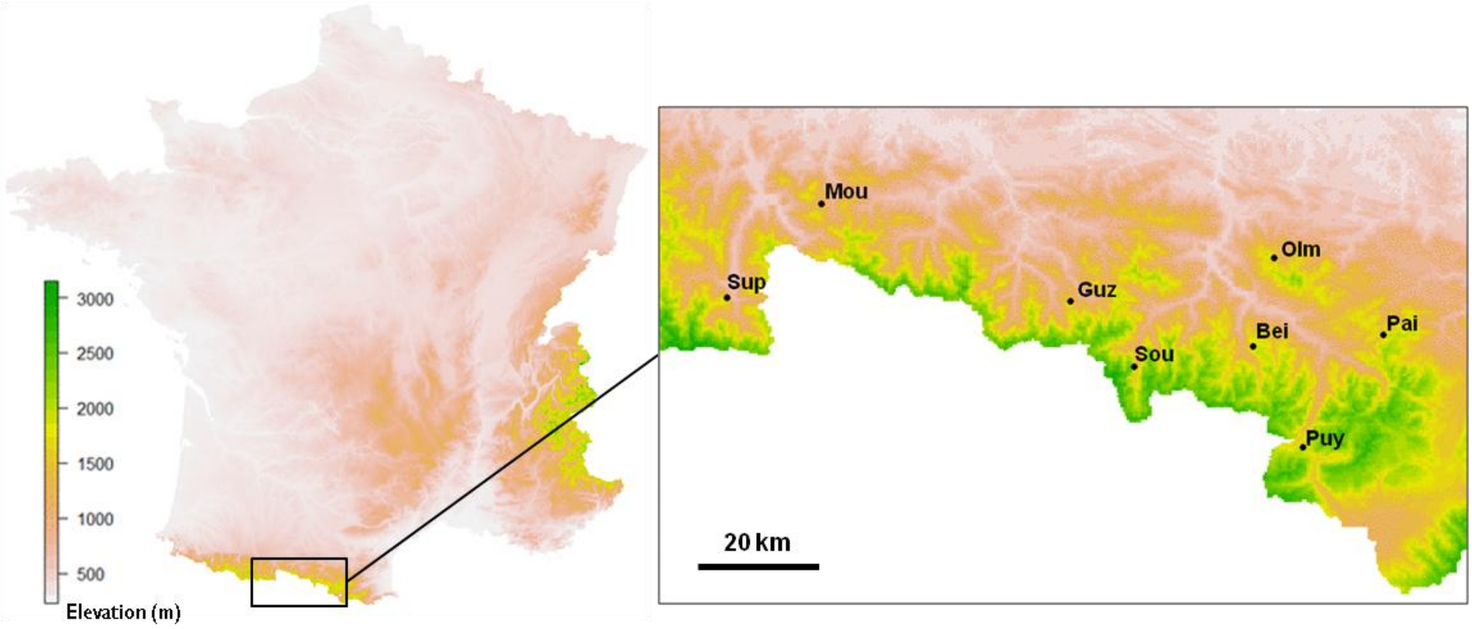

	Bei	Guz	Mou	Olm	Pai	Puy	Sou	Sup
Location	Beille	Guzet	Le Mourtis	Monts d'Olmes	Pailhères	Puymorens	Soulcem	Superbagnères
Coordinates (WGS84)	01°42'18.0"E 42°42'47.0"N	01°18'42.0"E 42°46'24.0"N	00°46'16.0"E 42°54'14.0"N	01°44'51.0"E 42°50'31.0"N	01°59'02.0"E 42°43'59.0"N	01°48'51.0"E 42°34'06.0"N	01°27'03.0"E 42°40'49.0"N	00°34'26.0"E 42°45'48.0"N
Elevation (m a.s.l.)	1940	1680	1670	1500	1900	1970	1510	1820

Supplementary Figure S1. Location of the study sites.

The map was generated with *R v3.0.2*[33] and *rgdal*[34] using the digital elevation model BD ALTI® MNT 500m from the French National Geographic Institute (IGN) (Open Licence).

Model	Year	Site	Air concentration (µg N m^{-3})		Dry deposition (mg N m^{-2} yr^{-1})		Wet deposition (mg N m^{-2} yr^{-1})		Total deposition (mg N m^{-2} yr^{-1})	
			NH_3	NO_2	Reduced N	Oxidized N	Reduced N	Oxidized N	Reduced N	Oxidized N
**EMEP**	2012	*Bei*	1.04	0.60	163	60	414	197	576	257
		Guz	1.34	0.63	224	49	549	225	773	274
		Mou	1.35	0.82	253	62	567	238	821	301
		Olm	0.92	0.67	176	82	421	225	597	307
		Pai	0.77	0.77	168	96	408	250	576	346
		Puy	1.06	0.67	156	57	409	199	565	255
		Sou	1.28	0.57	192	42	493	201	685	243
		Sup	1.24	0.59	197	44	481	197	678	241
	2013	*Bei*	0.97	0.58	156	51	432	190	588	241
		Guz	1.27	0.62	220	41	524	195	744	236
		Mou	1.27	0.81	245	55	608	256	853	311
		Olm	0.86	0.67	170	70	468	242	638	312
		Pai	0.72	0.78	161	82	450	263	611	345
		Puy	0.98	0.65	149	47	410	177	559	224
		Sou	1.19	0.55	187	35	468	167	655	201
		Sup	1.17	0.58	191	37	501	204	692	241
**CHIMERE**	2012	*Bei*	0.56	0.20	75	57	123	145	198	202
		Guz	0.84	0.18	105	53	132	134	236	188
		Mou	1.38	0.42	170	66	142	121	312	186
		Olm	1.08	0.60	128	77	127	130	255	207
		Pai	0.53	0.31	85	76	123	144	208	220
		Puy	0.38	0.63	44	60	102	138	145	198
		Sou	0.58	0.15	68	48	135	152	203	200
		Sup	0.83	0.22	102	51	129	139	231	190
	2013	*Bei*	0.53	0.20	72	48	118	141	190	189
		Guz	0.78	0.18	103	46	134	133	237	179
		Mou	1.32	0.42	169	60	165	139	334	199
		Olm	1.05	0.62	126	70	145	147	271	217
		Pai	0.49	0.31	84	67	123	147	207	214
		Puy	0.33	0.63	39	49	99	130	138	179
		Sou	0.52	0.15	62	38	124	142	186	180
		Sup	0.72	0.21	100	43	132	139	232	183

Supplementary Table S1. Modelled data from EMEP and CHIMERE after spatial interpolation. NH_3 and NO_2 air concentrations (µg N m^{-3}), annual reduced and oxidized dry, wet and total (dry + wet) N deposition (mg N m^{-2} yr^{-1}) estimated with the models EMEP and CHIMERE for the calendar years 2012 and 2013.

		Ascou	Aston	Augirein	Aulus	Bagneres	Cierp	Hospitalet	Olmes	Puymorens	Vicdessos
**Location**	Longitude	1°53'54"E	1°41'28"E	0°55'06"E	1°20'00"E	0°35'48"E	0°38'12"E	1°47'42"E	1°44'36"E	1°49'36"E	1°29'12"E
	Latitude	42°43'18"N	42°43'27"N	42°55'54"N	42°47'36"N	42°48'00"N	42°54'42"N	42°35'18"N	42°50'36"N	42°32'48"N	42°46'18"N
	Elevation (m a.s.l.)	1,120	1,781	631	733	620	500	1,425	1,500	1,620	750
**Measured precipitations (mm)**	2012	1,115	960	1,077	1,580	899	919	1,014	1,507	703	1,089
	2013	1,708	1,439	1,920	2,500	1,643	n.a.	1,400	2,214	n.a.	1,780
	June 2012 – May 2013	1,533	1,232	1,604	2,140	1,350	n.a.	1,191	1,961	n.a.	1,442
**EMEP precipitations (mm)**	2012	1,041	961	1,140	1,132	1,148	1,130	974	996	956	1,029
	2013	1,459	1,664	1,602	1,656	1,667	1,294	974	1,426	1,261	1,436
**CHIMERE precipitations (mm)**	2012	845	894	965	936	933	902	711	884	679	1,011
	2013	1,133	1,172	1,257	1,208	1,160	1,163	950	1,170	919	1,306

Supplementary Table S2. Precipitations.

Yearly precipitation accumulation (mm) at 10 meteorological stations close to the N deposition measurement sites. Measured precipitations for these stations were provided by Meteo-France. Modelled precipitation values from EMEP and CHIMERE were matched to these stations using the same methodology as for N concentration and deposition data (bilinear interpolation). Data are presented for the two calendar years of modelled data used in the study (2012-2013) and for the N measurement period of the study (June 2012 – May 2013) for measured precipitations. n.a.: measurements were lacking for some months of 2013 at these stations.
